# Phylogenetic reconsideration of *Myrmekiaphila* systematics with a description of the new trapdoor spider species *Myrmekiaphila tigris* (Araneae, Mygalomorphae, Cyrtaucheniidae, Euctenizinae) from Auburn, Alabama

**DOI:** 10.3897/zookeys.190.3011

**Published:** 2012-05-04

**Authors:** Jason E. Bond, Chris A. Hamilton, Nicole L. Garrison, Charles H. Ray

**Affiliations:** 1 Auburn University Museum of Natural History, Auburn University, Auburn, AL, 36849, USA

**Keywords:** New species, Integrative taxonomy, Molecular taxonomy, Southeastern United States, *Myrmekiaphila*, Cyrtaucheniidae, Euctenizinae

## Abstract

The trapdoor spider genus *Myrmekiaphila* currently comprises 11 nominal species. A recent molecular phylogenetic evaluation of the group identified a number of problems with respect to how species and species groups were delineated by Bond and Platnick in their 2007 taxonomic revision of the genus. We report herein the discovery of a new species, *Myrmekiaphila tigris* **sp. n.** The phylogenetic position of the species is evaluated using a molecular phylogenetic approach based on a set of mtDNA markers. Our preferred phylogenetic hypothesis supports the recognition of a new species and further highlights the need to more carefully investigate species boundaries within the genus. These results further indicate that palpal bulb morphology is rapidly evolving and has likely been a contributing factor in rendering a number of species paraphyletic with respect to the molecular data.

## Introduction

The genus *Myrmekiaphila* Atkinson, 1886 is a moderately diverse assemblage of trapdoor spiders distributed predominantly throughout the southeastern United States. Revised recently by Bond and Platnick (2007), it presently comprises 11 nominal species that range from Virginia southward to the Gulf Coast and extending as far west as central Texas. All known *Myrmekiaphila* species construct a silk-lined burrow, covered by a silken-soil trapdoor, from which they forage as sit and wait predators; many burrows are uniquely modified to include a side chamber that can be closed from the main chamber by a second trapdoor. As discussed by Bond and Platnick (2007) the familial placement of the genus has been somewhat problematic, however, molecular studies of mygalomorph phylogeny (Bond and Hedin 2006, Hedin and Bond 2006, and Bond et al. in review) place the genus in what is now recognized as the North American cyrtaucheniid subfamily Euctenizinae, to be elevated to family rank (Bond et al. in review).

**Figure 1.**
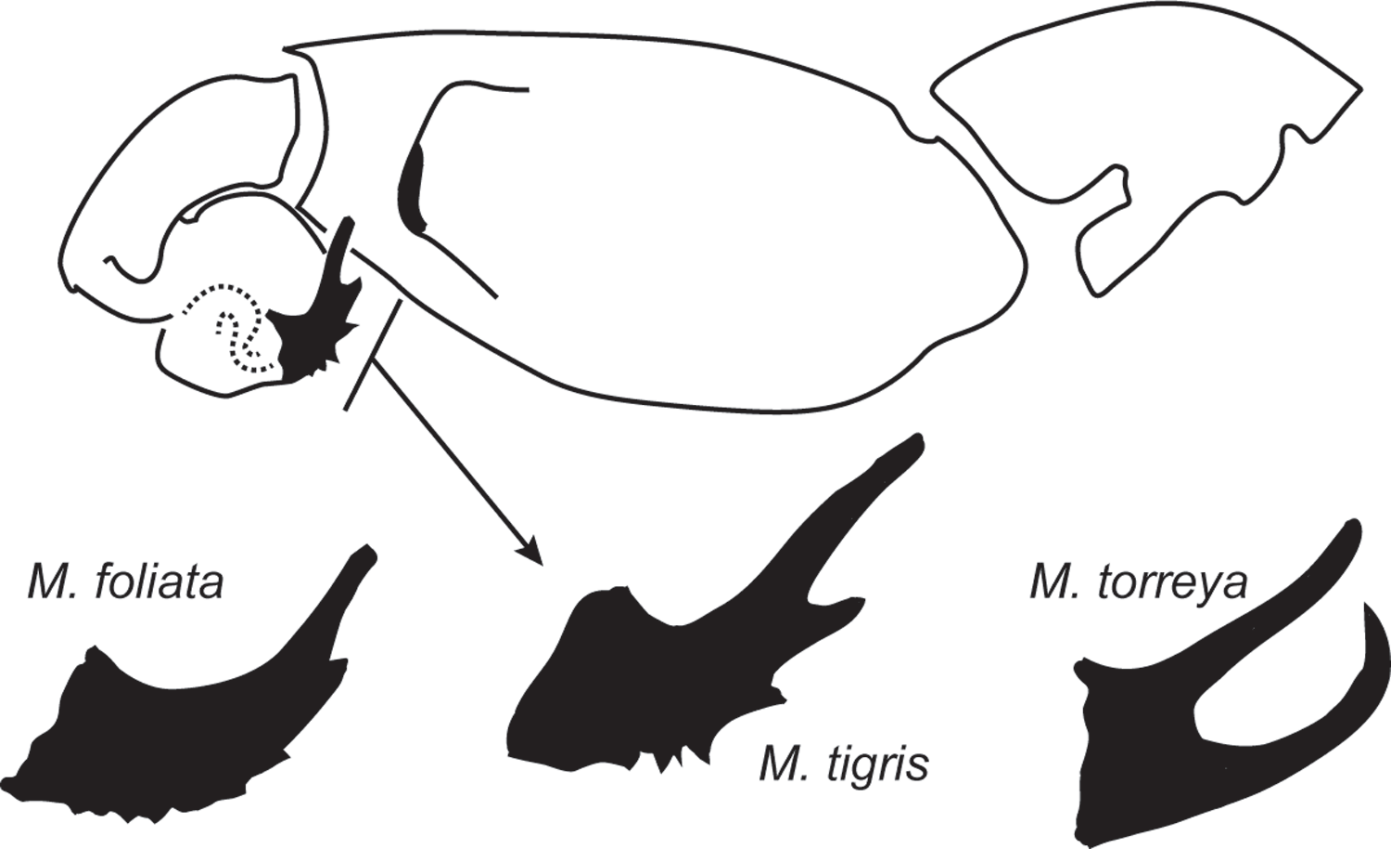
Line drawing of *Myrmekiaphila tigris* male pedipalp; distal aspect of palpal bulb shown for *Myrmekiaphila foliata*, *Myrmekiaphila tigris*, and *Myrmekiaphila torreya* for comparative purposes.

*Myrmekiaphila* species delimitation and diagnosis relies heavily on modifications of the male first walking leg – often termed the mating clasper – and the morphology of the palpal bulb. Mating clasper morphology is typically discriminated on the basis of unique spination patterns whereas palpal bulb modifications are often quite dramatic comprising relatively pronounced branched and serrated structures (Fig. 1). Such major differences in palpal bulb morphology stand in relatively stark contrast to the paucity of differentiation observed for palpal bulb structure in other related mygalomorph taxa (see Bond 2004, Stockman and Bond 2008). Despite the disparities in male morphology, female specimens are relatively homogenous with only subtle differences in spermathecae morphology rendering them nearly impossible to identify without the aid of molecular markers. Consequently, Bond and Platnick (2007) provided a morphological key only for male specimens.

On the basis of palpal differences, presence or absence of a second prong and serration patterns (Fig. 1 but also see Bailey et al. 2010, Figure 1), Bond and Platnick (2007) divided the genus into three species groups; they acknowledged at the time that these groupings were without phylogenetic support. A subsequent molecular based study of *Myrmekiaphila* species relationships by Bailey et al. (2010) indeed demonstrated these species groups were not monophyletic. Most notably the results of their analysis indicated that *Myrmekiaphila torreya* Gertsch & Wallace, 1936, a species with a dual pronged bulb, is paraphyletic with respect to *Myrmekiaphila coreyi* Bond & Platnick, 2007, a species with a single prong and diminutive serration. Consequently, the species “tree” (i.e., prior hypothesis of species boundaries) was in conflict with the gene tree. Bailey et al. (2010) hypothesized that palpal bulb evolution appeared to be quickly outpacing the evolutionary patterns conveyed by the mitochondrial and nuclear markers (incomplete lineage sorting) used to infer phylogeny and that it was unlikely that the putative *Myrmekiaphila torreya* lineages harbored cryptic diversity because of a general lack of geographical concordance among its lineages. This paper documents the discovery of a new *Myrmekiaphila* species that brings into question the hypothesis that incomplete lineage is the cause of the observed gene-tree species-tree incongruence. Rather, the study system may suffer from “bad taxonomy” (see Funk and Omland 2003). That is, that Bond and Platnick (2007) may have overlooked some of the morphological diversity contained within the group.

The focus of this paper is the description of a new species, *Myrmekiaphila tigris* Bond & Ray. This species was brought to the attention of the first author by C. Ray who collected numerous specimens during the winter months (Dec-Jan) of 2011/2012 from sidewalks and swimming pools located in the town of Auburn, Alabama in a moderately populated housing subdivision. Bond and Platnick (2007) had examined specimens from the vicinity of the type locality reported herein, but seemingly misidentified them as *Myrmekiaphila foliata* Atkinson, 1886. Examination of the newly acquired specimens and reexamination of the earlier collected material found these specimens to bear some likeness to *Myrmekiaphila foliata* but upon close inspection, notable differences in the palpal structure (discussed in the diagnosis of the species below) came to light. That said, given the putative widespread distribution of *Myrmekiaphila foliata* it would not be unreasonable to infer that the variation noted in these specimens simply represents a geographic variant of the species and thus recognition of new species is not necessarily warranted (i.e., if we ignore the fact that mygalomorph taxa are prone to species crypsis; see Bond and Stockman 2008). As such, it seemed prudent to consider an independent assessment of the hypothesized new taxon using molecular data before proposing a new name. The study reported here provides molecular evidence that warrants recognition of a new species, further documents the somewhat complicated relationships of species contained within the genus, and formally describes this newly recognized lineage as a nominal species.

## Materials and methods

Unique voucher numbers were assigned to all specimens (AUMS) and corresponding labels added to each vial. Collecting locality latitude/longitude data was georeferenced as described in Bond and Platnick (2007); georeferenced specimens from older museum labels given in brackets [ ] in the material examined section of the species description. All measurements were taken with a Leica MZ16.5 stereomicroscope equipped with a 10X ocular and ocular micrometer scale. Measurements were taken from the left appendage, usually in retrolateral view, using the highest magnification possible. Leg article measurements were taken as described in Bond and Platnick (2007). Illustrations were prepared using a Visionary Digital Imaging System (Ashland, VA). Photographs were recorded in multiple focal planes and assembled using the Zerene Stacker software package (Zerene Systems LLC, Richland, WA). Carapace and leg coloration are described using Munsell Soil Color Charts (Windsor, NY) and are given using the color name and hue value/chroma notation.

Species descriptions are formatted similarly to Bond (2004) and Bond and Platnick (2007) for consistency and comparative purposes. *Institutional abbreviations*: AMNH – American Museum of Natural History; CAU and AUMNH – Auburn University Museum of Natural History; CDF – personal collection of D. Folkerts, deposited in AUMNH; FMNH – Field Museum of Natural History. *Morphological abbreviations*: Cl/w (carapace length/width), STRl/w (sternum length/width), LBl/w (labium length/width), A/PER (anterior/posterior eye row), A/PME (anterior/posterior median eye), TSp/r and TSrd (male tibia I spines prolateral/retrolateral and retrolateral distal), PTw/l (male palpal tibia width/length), Bl (palpal bulb length), PT/TB3s (female patella/tibia III spines). Leg I article measurements are in the following order: femur, tibia, metatarsus, and tarsus; leg IV measurements are femur and tarsus only.

Protocols for sample, tissue, DNA extraction, polymerase chain reaction (PCR), and sequencing follow those outlined in Hendrixson and Bond (2007) and Bailey et al. (2010). Using those procedures we amplified for five exemplar specimens the 12S/16S mitochondrial DNA region using the PCR primers LR-J-12887 and SR-N-14612 (Simon et al. 1994). This mtDNA region was used by Bailey et al. (2010) in combination with a nuclear coding protein gene that showed a largely congruent phylogenetic pattern. Paratype specimens AUMS077, 089, 119 comprised a male and two female specimens collected from the type locality; AUMS095 is from a locality in the region, Tuskegee State Park. Specimen AUMS078 is a putative *Myrmekiaphila torreya* Gertsch & Wallace, 1936 juvenile specimen from the proximity of the *Myrmekiaphila tigris* type locality (outskirts of Auburn, AL, Lee County). The resulting fragment was sequenced using the PCR amplification primers plus an additional internal primer, SR-N-13xxxa (Bond and Stockman 2008). The fragments were assembled and edited using the computer program Geneious Pro v5.5.4 (Auckland, New Zealand). The final edited sequences (Genbank accession numbers JQ708211- JQ708215) were added to the existing *Myrmekiaphila* 12S/16S data matrix (Treebase accession S10740) and aligned to the existing alignment using the pairwise alignment tool in Mesquite (Maddison and Maddison 2009). Data partitions and model choice for Bayesian phylogenetic inference is outlined in Bailey et al. (2010).

Tree searches on the partitioned data set using Maximum Likelihood and Bayesian inference were conducted using RAxML ver. 7.2.8 (Stamatakis 2006) and MrBayes ver 3.1.2 (Huelsenbeck and Ronquist 2001; Ronquist and Huelsenbeck 2003), respectively. RAxML analyses comprised 1,000 random sequence addition replicates (RAS) using the commands “-# 1000” and “–m GTRGAMMA”. Bootstrap support values were calculated using the same search parameters with 1,000 replicates. Results from the bootstrap analysis were then applied to the best tree recovered from the RAS search. The MrBayes tree search comprised two independent runs of four simultaneous Markov Chain Monte Carlo (MCMC) chains. The Bayesian analyses were run for 20,000,000 generations with trees sampled every 1,000 generations; the first 25% were discarded as *burn-in*. Likelihood values for all post-analysis trees and parameters were evaluated for convergence and *burn-in* using the “sump” command in MrBayes and the computer program Tracer ver. 1.5 (Rambaut and Drummond; http://evolve.zoo.ox.ac.uk/software.html?id=tracer). Trees remaining after *burn-in* were used to calculate posterior probabilities using the “sumt” command.

## Data resources

Phylogenetic data sets (NEXUS and PHYLIP format) for all specimens evaluated in this study; locality data for *Myrmekiaphila tigris*.

The data underpinning the analysis reported in this paper were deposited on 25 April 2012 in the Dryad Data Repository at doi: 10.5061/dryad.pk24v (Bond et al. 2012) and at GBIF, the Global Biodiversity Information Facility, http://ipt.pensoft.net/ipt/resource.do?r=myr_dataset.

## Results and discussion

**Figure 2.**
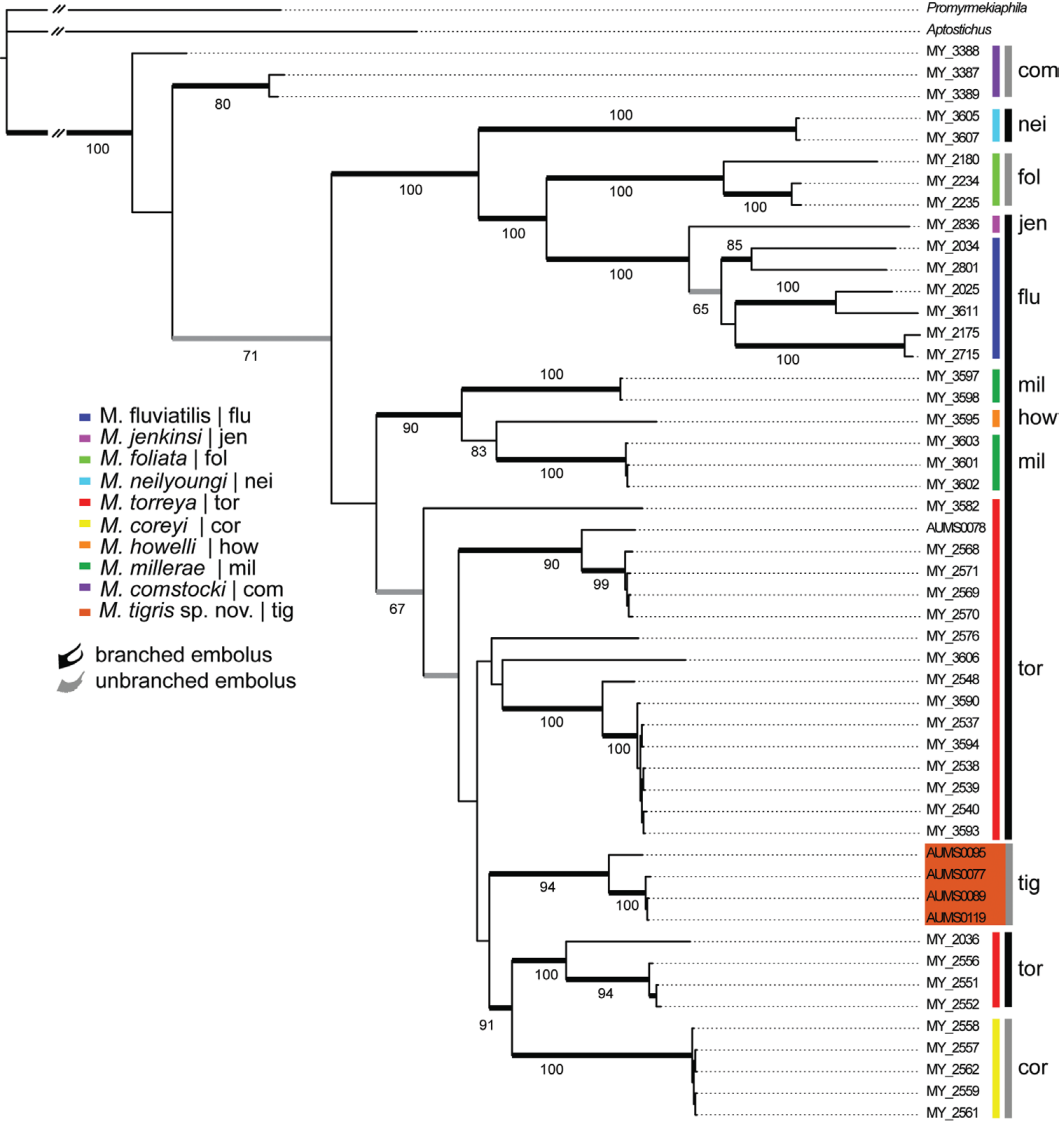
Preferred tree topology based on Bayesian analysis of the 12S/16S mitochondrial DNA data set. Color key and three-letter identifiers (inset) refer to species defined by Bond and Platnick 2007 along with the new species, *Myrmekiaphila tigris*, described in this study and palpal bulb condition (one-pronged vs. two-pronged). Thickened black branches indicated posterior probabilities > 0.95; gray branches denote values of 0.90–0.95. Numbers at nodes are bootstrap percentages from the maximum likelihood analysis conducted in RAxML.

The –ln likelihood value for the best scoring tree recovered from the RAxML analysis was -11,248.44. The harmonic and arithmetic means of the log likelihood values for all post *burn-in* topologies following MrBayes analyses were -11,307.70 and -11,364.56, respectively. Figure 2 summarizes the results of the phylogenetic analysis. The trees inferred from the both the Bayesian and likelihood analyses were largely congruent with respect to tree topology; bootstrap support values were less than posterior probabilities at some nodes.

The inferred phylogeny clearly indicates the hypothesized new species is not closely related to *Myrmekiaphila foliata* and is placed within the somewhat enigmatic *torreya* species group (sensu Bailey et al. 2010). Although its exact placement within the group as sister to a clade that includes both taxa with unbranched (*Myrmekiaphila coreyi*) and branched (*Myrmekiaphila torreya*, sensu lato) emboli lacks strong nodal support, its placement among taxa with divergent morphologies is strongly supported and unequivocal with respect to these data. Given the distinctiveness of the *Myrmekiaphila tigris* palpal bulb (Fig. 1), it would be impossible to confuse the new species with other members of the *torreya* clade. And, these specimens clearly do not share common ancestry with *Myrmekiaphila foliata*, thus do not represent a distinct geographic variant of that species. Based on these molecular data coupled with the distinctive, easily diagnosable morphology (Fig. 1), the recognition of a new species is clearly warranted. As discussed in the Taxonomy section below, specimens incorrectly identified as *Myrmekiaphila foliata* by Bond and Platnick (2007) are in need of reexamination.

The discovery of a second species with an unbranched embolus phylogenetically “embedded” within the *torreya* species group further complicates the systematics of the genus and generates more questions regarding species delimitation and the nature of the evolution of these somewhat enigmatic palpal bulb modifications. As discussed by Bailey et al. (2010), *Myrmekiaphila torreya* remains a paraphyletic species with respect to *Myrmekiaphila coreyi*, and now *Myrmekiaphila tigris*. Solutions to resolving the conflict between the gene tree and the taxonomy of this species group include recognizing all “basal” lineages as species (speciation by remote control sensu Templeton 1998) or collapsing the entire clade into a single species. Given the degree of morphological divergence contained within the lineage (sensu lato), considering the entire clade as one species is not sufficient and would overlook significant diversity. Alternatively, recognizing all of the lineages within the *torreya* group as species seems premature. First, as hypothesized by Bailey et al. (2010) *Myrmekiaphila torreya* paraphyly may simply reflect the rate at which genitalic change has occurred within the group as a consequence of sexual selection by female choice or sexual conflict thereby resulting in incomplete lineage sorting in the mtDNA data. The phylogeographic pattern reported here also fits with the hypothesis discussed by Bailey et al. (2010) that branched and unbranched palpal bulb conditions always occur where congeners are sympatric. Indeed, the phylogenetic position of specimen AUMS0078 (Auburn, AL, putative *Myrmekiaphila torreya* specimen) seems to suggest that *Myrmekiaphila torreya* and *Myrmekiaphila tigris* may be sympatric. However, the full extent and potential overlap of both species has not yet been thoroughly investigated. Nevertheless it does seem plausible that genitalic evolution by selection and/or as a consequence of classical character displacement could be outpacing the rate of molecular divergence. However, that hypothesis may continue to breakdown as specimens are more closely examined in light of the molecular data. The discovery of a new species coupled with the phylogenetic hypothesis further magnifies the complexities contained with the genus and may indicate that the lineages contained within the torreya clade be considered as five species. As such it seems clear that considerable work remains in terms of sampling more extensively (i.e. geographical, morphological, and molecular data); we simply lack sufficient data to properly delineate all of the species at this time. Consequently, considerable work remains if we are to gain an understanding of species boundaries and genitalic evolution within this interesting group of trapdoor spiders.

## Taxonomy

### Myrmekiaphila tigris

Bond & Ray sp. n.

urn:lsid:zoobank.org:act:1879EB33-3133-4A1A-96E5-5C2176A1AFB7

http://species-id.net/wiki/Myrmekiaphila_tigris

**Map 1.**
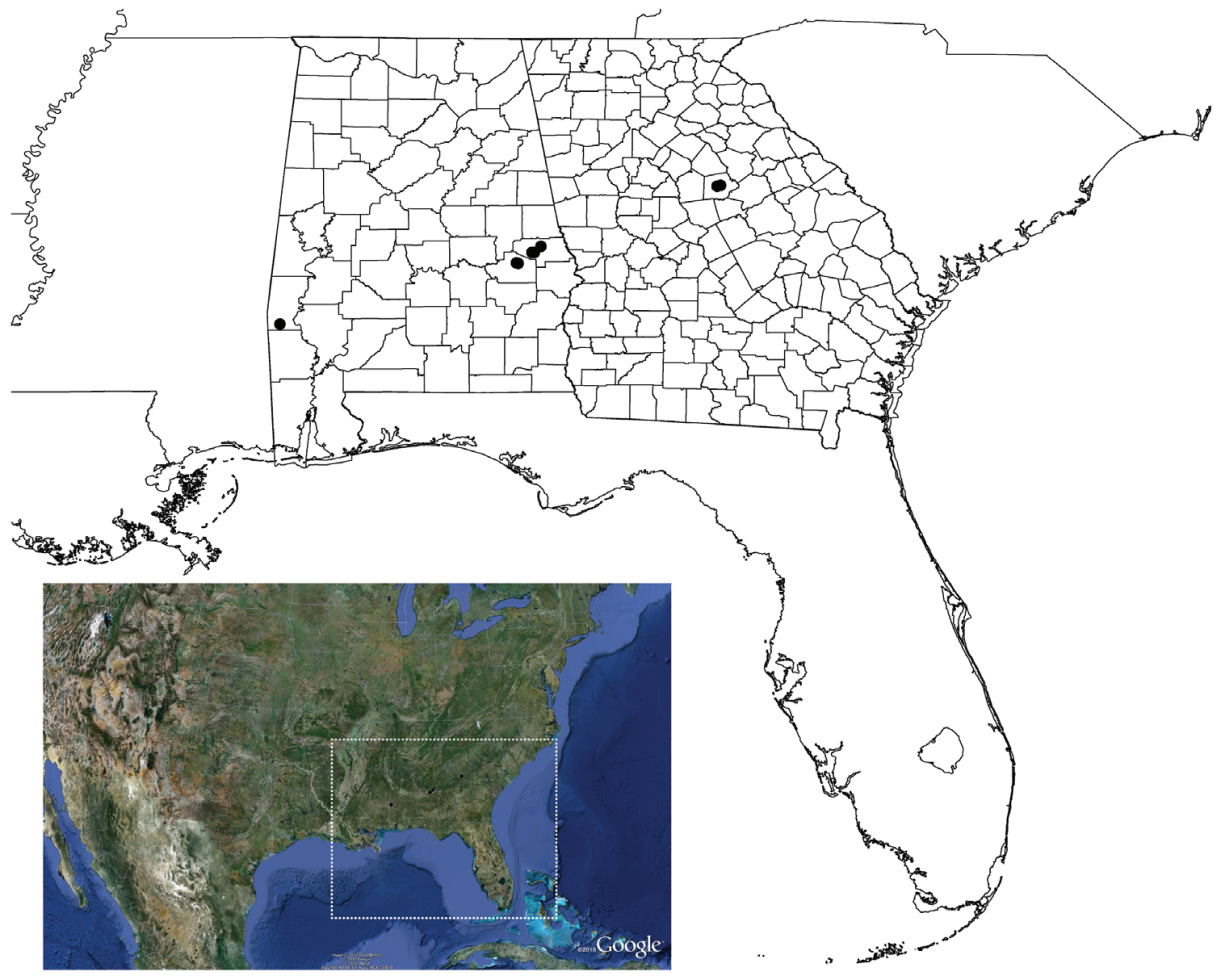
Geographic distribution of *Myrmekiaphila tigris*. County outlines for Alabama and Georgia are shown. Lower color inset, dotted line, shows extent of distribution map.

**Figures 3, 4.**
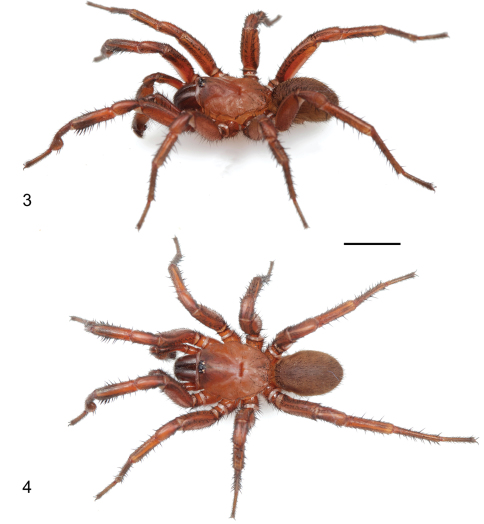
*Myrmekiaphila tigris* sp. n. male holotype specimen in life. **3** oblique view **4** dorsal view. Scale bar = 5mm.

Map 1 Figs 3 [Fig F5] 11

Myrmekiaphila foliata Atkinson, 1886 (misidentification): Bond and Platnick 2007: 9–10.

#### Common name: The Auburn Tiger Trapdoor Spider

#### Type material.

Male holotype (AUMS090), female paratype (AUMS089, from Alabama, Lee County, Auburn, along Grove Hill Road, forested area across from intersection with High Point Drive, 32.5786, -85.4543, 180m, coll. J. Bond 25.i.2012; additional male paratypes (AUMS077, 081–084) from same vicinity, coll. C. Ray i.2012. Male holotype and female paratype deposited in AUMNH; additional male paratypes deposited in AUMNH, AMNH and FMNH.

#### Additional material examined.

**ALABAMA: Choctaw Co.:** Silas [31.7654, -88.3290, MYR013], 19.ii.1912 (H. Smith, CUC), 1♂. **Lee Co.:** Opelika [32.6454. -85.3783, MYR133], 1.i.1985 (D. Folkerts, CDF), 1♂; 3.2km S Auburn along Wire Road [32.5776, -85.5246, MYR135], 24.ii.1974 (R. Skinner, CAU), 1♂; Auburn [32.6099, -85.4808, MYR124], (AMNH), 2♀, 1 juv., [MYR127], (N. Banks, C. Baker, MCZ), 1♀, [MYR132], 10.iv.1941 (AMNH), 3♀, [MYR137], 3.iii.1968 (W. Ivey, CAU), 1♂, [MYR139], 10.v.1975 (B. Muse, CAU), 1♀, [MYR288], 1.vi.1986 (G. Mullen, CAU), 1♀; Auburn, Grove Hill subdivision, 32.5786, -85.4543 (AUMS086–088, 091, 096–115, 117–119, 121, 123–127, 130–138), 180m, xii.2011-i.2012 (C. Ray, D. Held, J. Bond, N. Garrison, AUMNH), 41♂ 2♀, 5 juv. **Macon Co.:** Tuskegee National Forest, Wire Road, S Interstate 85 [32.4577, –85.6576, MYR138], 12.xii.1975 (Weatherby, Brooks, CAU), 1♀; Tuskegee National Forest, 32.4522, -85.6378 [AUMS094–095], 30.i.2012 (C. Hamilton, AUMNH), 2 juv. **Montgomery Co.:** McGus Station [MYR140], 24.x.1915 (H. Smith, CUC), 1♂. **GEORGIA: Putnam Co.:** no specific locality [33.3335, -83.3499, MYR040], 23.iv.1974 (W. Merrill, FSCA) 1♂; Eatonton [33.3268, -83.3885, MYR131], 18.iv.1974 (W. Merrill, FSCA), 1♂.

#### Etymology.

The specific epithet, the Latin name for tiger, is a noun taken in apposition and refers to the mascot of Auburn University.

#### Diagnosis.

Male palpal bulb morphology (Figs 7, 8) is similar to *Myrmekiaphila foliata* (Fig. 1, inset) but *Myrmekiaphila tigris* specimens have a longer sinuous embolus with an elongate sub distal tooth. Potentially sympatric *Myrmekiaphila torreya* males have two-pronged bulb whereas *Myrmekiaphila tigris* males have only a single prong. Males also appear to have a more robust palpal tibia with a larger retro-distal lateral ledge than in other species (Fig. 7). Females are much more difficult to definitively recognize from other species on the basis of morphological differences, however, the spermathecal base is considerably less wide than noted for *Myrmekiaphila torreya* (Bond and Platnick 2007) and central bulb has a more elongate stalk (Fig. 9). Also, male and female *Myrmekiaphila tigris* specimens tend to be larger in size than recorded for closely related *Myrmekiaphila coreyi*: *Myrmekiaphila tigris* Cl male> 6.50, female 7.36; *Myrmekiaphila coreyi* Cl male < 4.50, female < 6.00. Specimens are phylogenetically distinct as a monophyletic lineage exclusive of *Myrmekiaphila torreya*, *Myrmekiaphila coreyi*, and *Myrmekiaphila foliata* (Fig. 2). Known only from central Alabama and Georgia.

#### Description of male holotype.

**Figures 5–9. F5:**
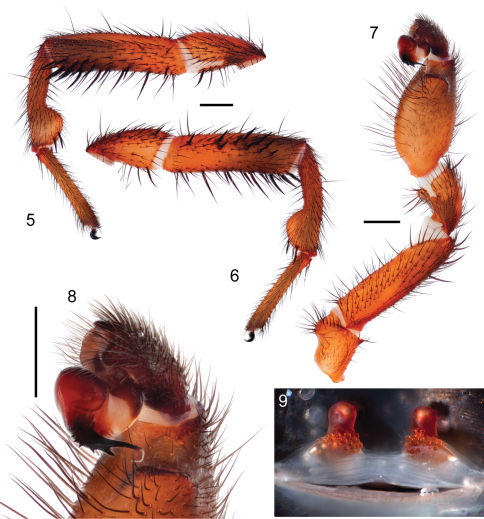
*Myrmekiaphila tigris* sp. n. male holotype and female paratype. **5, 6** 1^st^ walking leg of male, left side retrolateral and prolateral view **7** pedipalp, retrolateral view **8** palpal bulb **9** cleared spermathecae. Scale bars = 1mm.

*Specimen preparation and condition*. Specimen collected live from burrow, preserved in 80%. Pedipalp, leg I left side removed, stored in vial with specimen. *General coloration*. Carapace dark red 2.5YR 3/6; legs, chelicerae darker in color, dusky red 10R 3/4. Abdomen dark brown 7.5YR 3/2 with broad faint dusky stripes posteriorly dorsal (Figs 3, 4), ventrum spinnerets pale yellow. *Cephalothorax*. Carapace 6.56 long, 5.63 wide, hirsute with thin black short setae, stout black bristles along fringe; surface smooth, pars cephalica elevated. Fringe, posterior margin with black bristles. Foveal groove deep, straight. Eyes only slight elevated. AER slightly procurved, PER slightly recurved. PME slightly larger in diameter than AME. Sternum moderately setose, STRl 3.72, STRw 3.35. Posterior sternal sigilla large, irregularly shaped, nearly contiguous, anterior sigilla pairs small, oval, marginal. Chelicerae with distinct anterior tooth row comprising 11 teeth, posterior margin with single row small denticles. Palpal endites with patch of small cuspules on proximal, inner margin, labium lacks cuspules, LBw 1.19, LBl 0.70. Rastellum consists of 4 stout spines on distinct mound. *Abdomen*. Setose, heavy black setae intermingled with fine black setae. *Legs*. Leg I: 5.85, 2.88, 4.25, 3.41, 2.84; leg IV: 6.00, 3.25. Light scopulae on tarsi, metatarsi legs I, II. Tarsus I with single, slightly staggered row of 9 trichobothria. Leg I spination pattern illustrated in Figures 4, 5; TSp 12, TSr 10, TSrd 2. *Pedipalp*. Articles stout, lacking distinct spines (figs 6, 7). PTw 1.50, PTl 3.00, Bl 1.24. Distinct, elongate ledge on distal-retrolateral aspect tibia. Embolus stout, tapering sharply towards tip, with serrations, elongate distal tooth (Fig. 8).

**Variation (8).** Cl 6.81–10.30, 7.56±0.41; Cw 5.40–8.08, 6.08±0.30; STRl 3.80–5.25, 4.14±0.17; STRw 3.38–4.85, 3.71±0.17; LBw 1.02–1.57, 1.21±0.07; LBl 0.56–0.70, 0.64±0.02; leg I: 5.35–8.32, 6.29±0.31; 4.60–5.75, 4.73±0.16; 3.25–4.85, 3.75±0.18; 2.88–4.20, 3.14±0.16; leg IV: 5.75–8.88, 6.52±0.35; 3.09–4.45, 3.48±0.16; PTl 3.00–4.32, 3.27±0.15; PTw 1.56–2.13, 1.68±0.07; Bl 1.20–1.60, 1.30±0.04; TSp 12–21, 15.25±1.01; TSr 10–14, 11.75±0.41; TSrd 1–2, 1.88±0.13.

#### Description of female paratype.

**Figures 10, 11.**
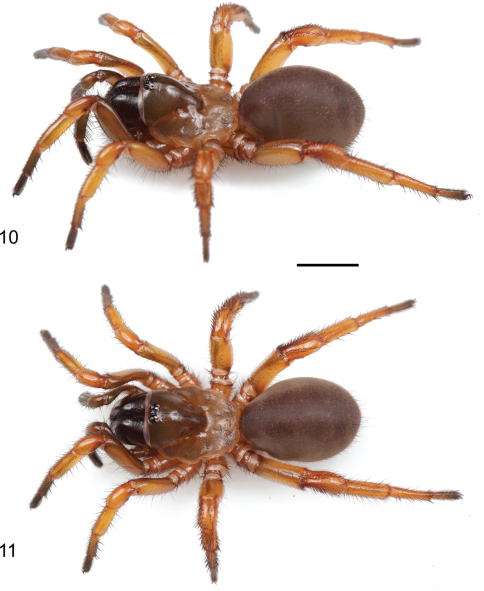
*Myrmekiaphila tigris* sp. n. female paratype specimen in life. **10** oblique view **11** dorsal view. Scale bar = 5mm.

*Specimen preparation and condition*. Female collected live from burrow, preserved in same manner as male holotype. Genital plate removed, cleared in trypsin, stored in microvial with specimen. *Color*. Carapace dark reddish gray 2.5YR 3/1; legs, chelicerae, dark reddish brown 2.5YR 3/4. Abdomen reddish black dorsally 2.5YR 2.5/1 faint dusky bands dorsally; ventrum, spinnerets pale yellow (figs 10, 11). *Cephalothorax*. Carapace 7.36 long, 6.38 wide, generally glabrous, few thin setae, pars cephalica elevated. Fringe lacks setae. Foveal groove deep, slightly procurved. Eye group slightly elevated on very low mound. AER slightly procurved, PER slightly recurved. PME-AME subequal in diameter. Sternum widest at coxae II/III, moderately setose, STRl 4.60, STRw 4.08. Three pairs of sternal sigilla anterior pairs moderate size, oval, positioned marginally, posterior pair larger, irregularly shaped, nearly contiguous. Chelicerae anterior tooth row armed with 10 teeth with single posterior margin denticle row. Palpal endites with 47 cuspules concentrated at the inner promargin posterior heel; labium with 5 cuspules, LBw 1.46, LBl 0.97. Rastellum consist of 10 very stout spines positioned on distinct mound. *Abdomen*. Moderately setose, posterior median spinnerets reduced in size. *Walking legs*. Anterior two pairs noticeably more slender than posterior pairs. Leg I 16.39 long. Tarsus I with single staggered row of 10 trichobothria. Legs I, II with moderately heavy scopulae on tarsi, metatarsi. PT3s 14, TB3s 9. Rudimentary preening comb on retrolateral distal surface, tarsus - metatarsus joint metatarsus III, IV. *Spermathecae*. Two simple spermathecal bulbs, moderately elongate neck, arranged on low subtriangular base (fig. 9).

#### Variation.

Females known only from three specimens.

#### Distribution and natural history.

Known from central Alabama, counties of Choctaw, Lee, Macon, and Montgomery, and the piedmont region of Georgia, Putnam County (Map 1). The type locality comprises primarily young second growth mixed deciduous forest located at the transition from the Piedmont to Coastal Plan physiographic region. The population known from the additional material in western Alabama is located in Coastal Plain Province. Specimens were found to be presumably syntopic with *Cyclocosmia*, *Antrodiaetus*, *Ummidia*, and possibly *Myrmekiaphila torreya* (collected from the region). Male specimens were collected from swimming pools and wandering on warm, damp mornings during the months of December and January. Females were collected from 6–8 cm deep burrow, some with below-ground side chambers with a trapdoor.

#### Genbank accession numbers.

JQ708212–JQ708215

## Supplementary Material

XML Treatment for Myrmekiaphila tigris

## Appendix 1

Locality data for *Myrmekiaphila tigris*.

**Explanation note:** Locality data (CSV format) for *Myrmekiaphila tigris* specimens (doi: 10.3897/zookeys.190.3011.app1). File format: Microsoft Excel comma delimited.

**Copyright notice:** This dataset is made available under the Open Database License (http://opendatacommons.org/licenses/odbl/1.0/). The Open Database License (ODbL) is a license agreement intended to allow users to freely share, modify, and use this Dataset while maintaining this same freedom for others, provided that the original source and author(s) are credited.
